# Learning-induced synaptic potentiation in implanted neural precursor cell-derived neurons

**DOI:** 10.1038/srep17796

**Published:** 2015-12-04

**Authors:** Kyungjoon Park, Hwon Heo, Ma Eum Han, Kyuhyun Choi, Jee Hyun Yi, Shin Jung Kang, Yunhee Kim Kwon, Ki Soon Shin

**Affiliations:** 1 Department of Biology, Department of Life and Nanopharmaceutical Sciences, Kyung Hee University, Seoul, Republic of Korea; 2 Department of Molecular Biology, Sejong University, Seoul, Republic of Korea

## Abstract

Neuronal loss caused by neurodegenerative diseases, traumatic brain injury and stroke results in cognitive dysfunctioning. Implantation of neural stem/precursor cells (NPCs) can improve the brain function by replacing lost neurons. Proper synaptic integration following neuronal differentiation of implanted cells is believed to be a prerequisite for the functional recovery. In the present study, we characterized the functional properties of immortalized neural progenitor HiB5 cells implanted into the rat hippocampus with chemically induced lesion. The implanted HiB5 cells migrated toward CA1 pyramidal layer and differentiated into vGluT1-positive glutamatergic neurons with morphological and electrophysiological properties of endogenous CA1 pyramidal cells. Functional synaptic integration of HiB5 cell-derived neurons was also evidenced by immunohistochemical and electrophysiological data. Lesion-caused memory deficit was significantly recovered after the implantation when assessed by inhibitory avoidance (IA) learning. Remarkably, IA learning preferentially produced long-term potentiation (LTP) at the synapses onto HiB5 cell-derived neurons, which occluded paring protocol-induced LTP *ex vivo*. We conclude that the implanted HiB5 cell-derived neurons actively participate in learning process through LTP formation, thereby counteracting lesion-mediated memory impairment.

Neuronal death is a common pathological phenomenon in traumatic or neurodegenerative brain diseases. Implantation of neural precursor cells (NPCs) to replace lost neurons offers promising therapeutic strategy yielding functional recovery from the disease states. In various animal models, such as Alzheimer’s disease, Parkinson’s disease, multiple sclerosis and stroke, functional recovery by NPC implantation into damaged brain regions has been documented^1^,^2^,^3^,^4^. Implanted NPCs differentiate into functional mature neurons that integrate into existing neuronal circuits^5^,^6^. Therefore, damaged neural circuits can be reconstructed, albeit partially, by implanted NPC-derived neurons to restore brain functions. Functional recovery may require functional synaptic connections of implanted NPC-derived neurons with existing endogenous neurons^7^,^8^,^9^.

The hippocampus is a brain region critical for the acquisition, consolidation and retrieval of declarative memory^10^. Therefore, neuronal loss in the hippocampus causes memory impairment. Functional recovery from the deficit by implanted NPCs has been recently demonstrated in rodent models of hippocampal damage^11^,^12^,^13^. However, the mechanisms of the functional recovery remain unclear. In physiological conditions, changes in synaptic efficacy (i.e., long-term potentiation (LTP) and long-term depression) have been widely accepted as a cellular mechanism for learning and memory. The causal link between these processes and memory has been demonstrated in the hippocampus as well as other brain areas using different behavioral paradigms^14^,^15^. However, it has not been examined whether implanted NPC-derived neurons actively participate in learning and memory by modifying their own synaptic efficacy.

In the present study, we show that implanted immortalized NPCs, HiB5 cells, into the hippocampus migrated and differentiated into functional glutamatergic pyramidal neurons. In addition, they integrated into the hippocampal neural circuit. Implantation improved memory impairment in the rat model of hippocampal lesion. Remarkably, we demonstrate for the first time that the implanted NPC-derived neurons can display learning-induced LTP. Therefore, we conclude that implanted NPC-derived neurons actively participate in learning process through LTP formation, thereby contributing to the functional recovery from hippocampal damage.

## Results

### Morphological and electrophysiological characteristics of the implanted neuronal precursor cells

**Figure 1:**
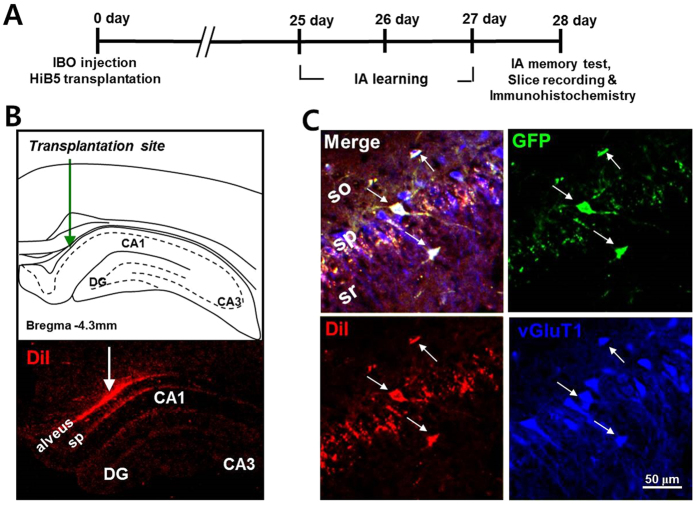
Implanted HiB5 cells differentiate into glutamatergic neurons in the hippocampus of IBO lesion rats. (**A**) A schematic diagram depicts the overall experimental schedule for the present study. Numerals indicate days after surgery. IBO and HiB5 cells were stereotaxically injected into the entorhinal cortex and alveus of the dorsal hippocampus, respectively. Rats were allowed to recover for 24 days and then inhibitory avoidance (IA) learning was performed for 3 days. The next day, IA memory test, slice recording and immunohistochemistry were conducted. (**B**) Illustration of hippocampal slices showing implantation site of HiB5 cells in the dorsal hippocampus (upper panel) and migration of DiI-positive implanted HiB5 cells toward CA1 pyramidal layer (bottom panel). (**C**) Representative images of implanted HiB5 cell-derived neurons in CA1 pyramidal layer 28 days after implantation. HiB5 cells identified by immunofluorescence with an antibody to GFP (green) and DiI (red) gave rise to vGluT1-positive neurons (blue) in the CA1 pyramidal layer of the IBO-lesion rat hippocampus. So, stratum oriens; py, stratum pyramidale; sr, stratum radiatum.

The entorhinal cortex is a primary source of synaptic inputs to the hippocampus and influences learning and memory^16^. Lesion in the entorhinal cortex has been known to result in progressive cell loss in the hippocampus, thereby producing cognitive dysfunction^17^,^18^,^19^,^20^. Therefore, injection of ibotenic acid (IBO) into the entorhinal cortex generates neuronal loss in the hippocampus as well as the entorhinal cortex. In the present study, exploiting the IBO-lesion rat model, we examined whether neuronal precursor cells implanted in the hippocampus can differentiate into functional neurons and integrate into the existing neural network. The overall experimental schedule is presented in Fig. 1A. IBO was bilaterally injected targeting at the entorhinal cortex. HiB5 cells were bilaterally injected into the alveus of dorsal hippocampal area (Fig. 1B, upper panel). HiB5 cells are rat hippocampal precursor cells immortalized with temperature-sensitive mutant ts A58/U19 of SV40 T-antigen, rendering HiB5 cells to grow continuously at 33 °C, but not at 37 °C, the body temperature of rodents^21^. Therefore, HiB5 cells stop proliferating upon implantation into the rat brain. The implanted HiB5 cells were labeled with both GFP (using GFP-adenovirus) and DiI-C18-(3) (DiI) for tracking the implanted HiB5 cells.

**Figure 2:**
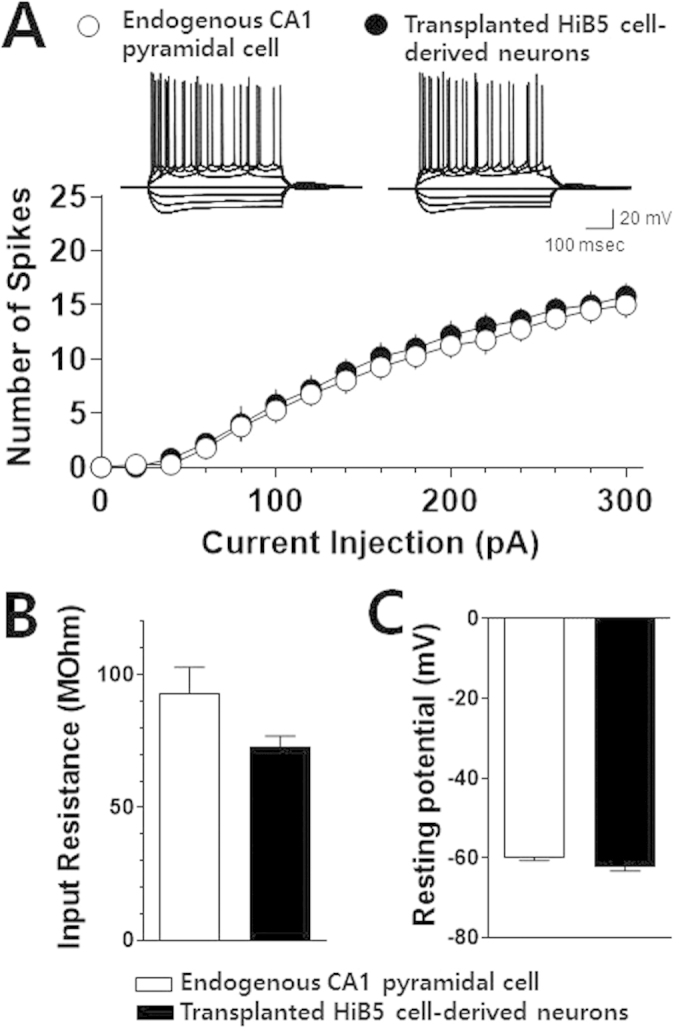
Intrinsic membrane properties of the implanted HiB5 cell-derived neurons are comparable to those of endogenous CA1 pyramidal cells. (**A**) Action potentials in the implanted HiB5 cell-derived neurons and endogenous CA1 pyramidal cells. Inset shows representative recording traces in a current-clamp mode elicited by current steps from HiB5 cell-derived neurons and endogenous CA1 pyramidal cells. The current steps were from −300 pA to +300 pA in +100-pA increments. Graph shows average numbers of action potentials evoked by a series of depolarizing current steps from the implanted HiB5 cell-derived neurons and endogenous CA1 pyramidal cells. Values are mean ± SEM. (**B**) Input resistance in the implanted HiB5 cell-derived neurons and endogenous CA1 pyramidal cells. 92.98 ± 9.77 MΩ for HiB5 cell-derived neurons, n = 5; 72.68 ± 4.23 MΩ for endogenous pyramidal cells, n = 4; P > 0.05, unpaired Student’s t-test. (**C**) Resting membrane potential in the implanted HiB5 cell-derived neurons and endogenous CA1 pyramidal cells. –62.28 ± 0.96 mV for HiB5 cell-derived neurons, n = 5; –59.95 ± 0.66 mV for endogenous pyramidal cells, n = 4; P > 0.05, unpaired Student’s t-test.

Twenty-eight days after surgery, the implanted DiI-positive HiB5 cells were found in the hippocampus including CA1 stratum pyramidale (sp, Fig. 1B, bottom panel) besides the injection site. About 13% of the injected HiB5 cells survived 28 days after implantation (see Supplementary Fig. S1 on line). Furthermore, the majority of the implanted cells were NeuN^+^ neurons (69.88 ± 3.65%, see Supplementary Fig. S2 on line). We also found that most HiB5 cells found in CA1 stratum pyramidale were vGluT1-positive neurons (Fig. 1C), indicating that a great portion of implanted HiB5 cells give rise to glutamatergic neurons. In fact, the immunohistochemical observation showed that a half of the implanted HiB5 cells (48.21 ± 1.76%) differentiated into glutamate-positive excitatory neurons (see Supplementary Fig. S2 on line). Using whole-cell patch-clamp recordings in brain slices, we further confirmed that these HiB5 cell-derived vGluT1-positive cells are also electrophysiologically mature. Current-clamp recordings from GFP (green) and DiI (red) double-positive cells revealed action potentials triggered by depolarizing current steps and they were comparable to those of nearby endogenous pyramidal cells (Fig. 2A). The recordings also revealed that input resistance (Fig. 2B) and resting membrane potential (Fig. 2C) were not different between the groups. Collectively, implanted HiB5 cell-derived neurons had the similar intrinsic electrical properties of endogenous CA1 pyramidal cells.

**Figure 3:**
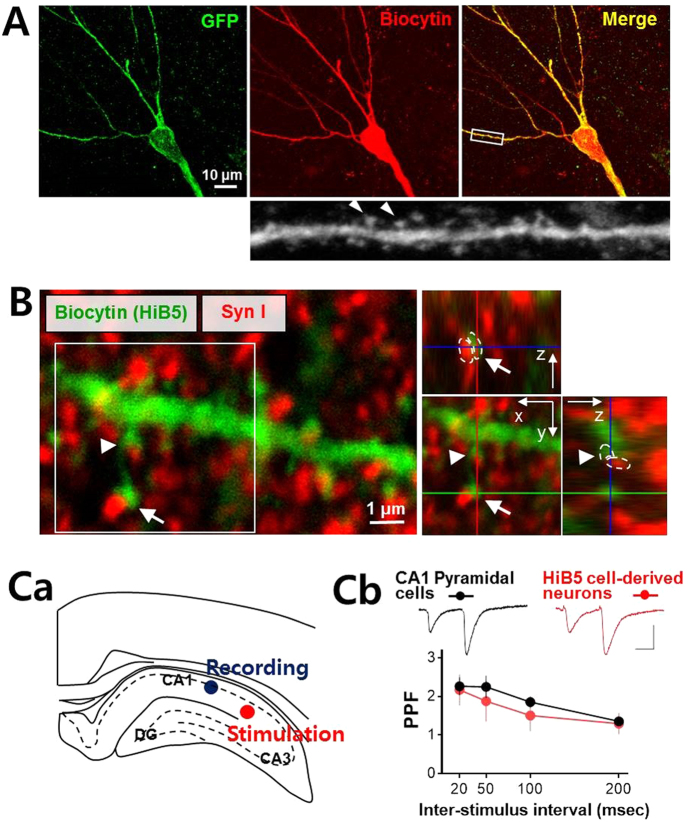
Implanted HiB5 cell-derived neurons functionally integrate into hippocampal neural circuits. (**A**) Dendritic architecture of the implanted HiB5 cell-derived neurons in CA1. Recorded HiB5 cell-derived neurons were retrospectively visualized by immunofluorescence staining for GFP (green) and biocytin (red). Mushroom-shaped dendritic spines (arrow heads) were clearly seen in the enlargement of the secondary dendrite boxed in the merged image. (**B**) Apposition of biocytin-positive spines of the HiB5 cell-derived neurons (green) and synapsin I-positive presynaptic terminals (red). The z-stack reconstruction of the boxed area clearly demonstrates the synapse formation on dendritic spines of the implanted HiB5 cell-derived neurons (arrow and arrowhead). (**C**) Paired-pulse facilitation at synapses in the HiB5 cell-derived neurons. Ca illustrates stimulation and recording sites in coronal brain slices. Synaptic responses were evoked in neurons in CA1 pyramidal layer by stimulation of Schaffer collateral pathway. Cb shows paired-pulse ratio (PPR, EPSC2/EPSC1) from the endogenous CA1 pyramidal cells and the implanted HiB5 cell-derived neurons. The insets show representative electrophysiological traces averaged 5 consecutive EPSCs evoked by paired pulses (50 ms inter-stimulus interval) in an endogenous CA1 pyramidal cell and a implanted HiB5 cell-derived neuron (scale bars: 25 ms and 50 pA).

For a detailed morphological analysis of the recorded HiB5 cell-derived neurons, biocytin was included in the pipet solution. Following the electrophysiological recording, the recorded cells were processed for immunohistochemistry for biocytin. *Post hoc* immunohistochemical staining showed clear colabeling of GFP and biocytin and revealed detailed architecture of dendrites in the implanted HiB5 cell-derived neurons; the multiply branched dendrites harbored a number of mushroom-shaped spines (arrowheads, Fig. 3A). We next sought to determine whether synapses were formed between host neurons and the implanted HiB5 cell-derived neurons. Synapsin I is a presynaptic phosphoprotein^22^ and antibodies to this protein has been found to be useful in labeling the vast majority of cortical synapses with minimal labeling at non-synaptic loci^23^. Therefore, we localized the synapses between endogenous cells and HiB5 cell-derived neurons using the immunofluorescence of synapsin I and biocytin. As shown in Fig. 3B, biocytin-labeled dendritic spines of HiB5 cell-derived neurons were closely apposed to the presynaptic protein synapsin I (arrows). This observation suggests that the functional synapses might be built on the implanted HiB5 cell-derived neurons.

To provide direct evidence for the functional synapse formation, electrophysiological recordings were performed. While recording from GFP- and DiI-double positive HiB5 cell-derived neurons located in CA1 stratum pyramidale, we applied paired-pulse stimulation on Schaffer collateral pathway (Fig. 3Ca). Schaffer collaterals are the axons of CA3 pyramidal cells that project to CA1 pyramidal cells and transfer information from CA3 to CA1^24^,^25^. Paired-pulse stimulation generated evoked EPSCs in HiB5 cell-derived neurons that exhibited paired-pulse facilitation (PPF) similar to that in endogenous CA1 pyramidal cells (Fig. 3Cb). Two-way ANOVA indicates that the level of PPF was not significantly different between the HiB5 cell-derived neurons and endogenous CA1 pyramidal cells (cell type F_(1, 15)_ = 2.96, P = 0.1058; cell type × inter-stimulus interval F_(3, 45)_ = 1.25, P = 0.3015; inter-stimulus interval F_(3, 45)_ = 29.83, P < 0.0001; endogenous CA1 pyramidal cells, n = 6; HiB5 cell-derived neurons, n = 11). This finding suggests that the implanted HiB5 cell-derived neurons functionally integrate into an existing hippocampal neural circuit. On the whole, it appears that implanted HiB5 cell-derived neurons can adopt similar neuronal functionalities to endogenous CA1 pyramidal cells. In this regard, we next questioned whether the implantation of HiB5 cells can restore memory deficit found in IBO-lesion rat model.

### Behavioral effect of HiB5 implantation in IBO-lesion rat model

**Figure 4:**
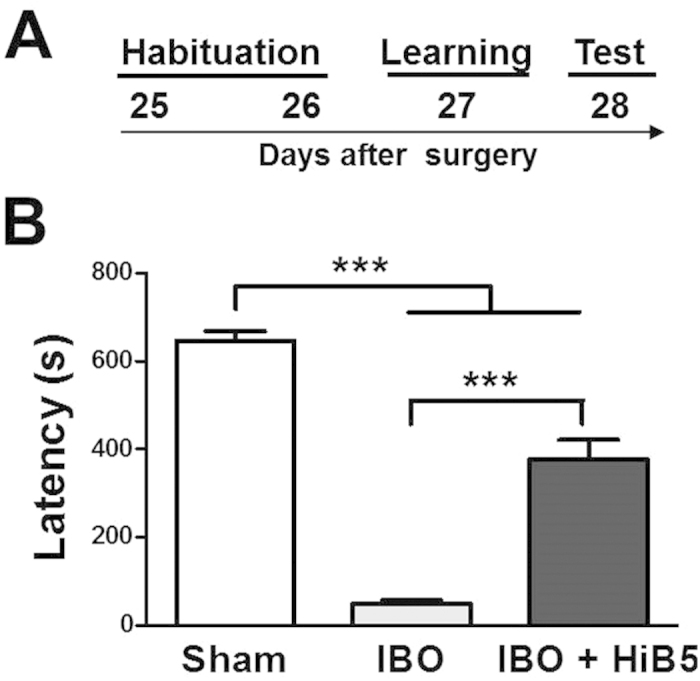
Implantation of HiB5 cells counteracts a learning deficit caused by IBO lesion. (**A**) Schematic diagram of IA learning schedule. Numerals indicate days after surgery. (**B**) Escape latency from the lighted chamber was recovered by HiB5 implantation in the IBO-lesioned rats. The latencies were measured in saline-injected rats (Sham), IBO-lesion rats (IBO) and HiB5 cell-implanted IBO-lesion rats (IBO + HiB5). ***Significantly different (P < 0.001), one-way ANOVA with Newman-Keuls posttest. The values are mean ± SEM.

Exploiting inhibitory avoidance (IA) learning, now we tested the possibility that implantation of HiB5 cells is capable of improving the behavioral deficit observed in the IBO-lesion animal model. Time schedule for the IA learning is shown in Fig. 4A, and the detailed experimental procedure was described in the materials and methods. IA memory was measured as the tendency for the animals to avoid the dark chamber after the training. As shown in Fig. 4B, the IBO-lesioned rats (IBO) were unable to learn avoiding foot shock-associated dark chamber. This learning deficit was remarkably improved by implantation of HiB5 cells (IBO + HiB5) (F_(2, 40)_ = 37.60, P < 0.0001, one-way ANOVA): The latency time for the IBO group was drastically decreased compared to the saline-injected sham (Sham) group (IBO: 49.62 ± 7.62 s, n = 13; Sham: 647.10 ± 20.72 s, n = 7; P < 0.001, *post hoc* Newman-Keuls test), but it was substantially recovered by HiB5 cell implantation (IBO + HiB5: 378.10 ± 42.60 s, n = 23, P < 0.001, *post hoc* Newman-Keuls test). The data indicate that the implantation of HiB5 cells improved the learning deficit observed in the IBO-lesion animal model.

### Participation of the implanted HiB5 cell-derived neurons in learning process

**Figure 5:**
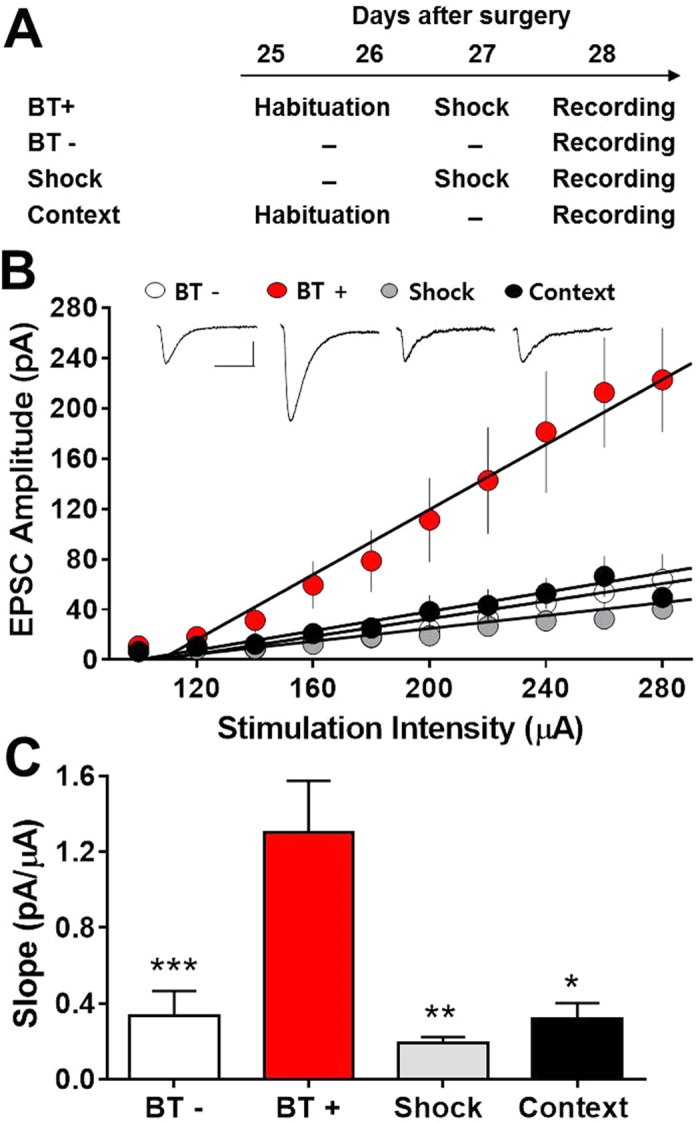
IA learning produces LTP at synapses onto the implanted HiB5 cell-derived neurons. (**A**) Schematics for behavioral treatments: IA learning (BT+), homecage control (BT−), shock control (Shock) and context control (Context). Numerals indicate days after surgery. (**B**) Input-output relationships of EPSCs for implanted HiB5 cell-derived neurons in slices from rats with different behavioral treatments as shown in A. Insets demonstrate averaged current traces of five consecutive EPSC responses with input stimulations of 280 μA. Scale bars, 40 ms and 40 pA. (**C**) Comparison of the slopes calculated from the input-output curves shown in B. The slope from BT+ rats was significantly greater than that from BT−, shock or context control rats. *P < 0.05, **P < 0.01, ***P < 0.001, one-way ANOVA with Newman-Keuls posttest (compared with BT+). Values are mean ± SEM.

It has been shown that IA learning leads to synaptic strengthening in CA1 of the dorsal hippocampus^26^ and requires synaptic transmission from CA3 to CA1^27^. We found that the implanted HiB5 cells differentiated into morphologically and electrophysiologically mature pyramidal neurons in CA1. In addition, IBO-lesioned rats that received HiB5 cells implantation exhibited the restoration of learning deficit upon IA learning. Therefore, we questioned whether IA learning causes changes in synaptic efficacy in the implanted HiB5 cell-derived neurons. To address this question, the input-output relationships for the excitatory postsynaptic current (EPSC) amplitude as a function of stimulus intensity were compared in four groups: BT+, BT−, Shock and Context. BT+ is a group of rats subjected to IA learning. Control groups were either homecage control (BT−), given a foot shock in the dark chamber and immediately removed from the apparatus (shock control, Shock), or habituated for 2 days without shock training (context control, Context). Experimental schedule is shown in Fig. 5A. Recordings were made from hippocampal slices prepared 1 day after the behavioral treatments and EPSCs were evoked by stimulating Schaffer collateral pathway. Input-output relationships for the EPSC amplitude were shown in Fig. 5B. The slopes of the linear fits to the data points in each neuron were obtained and they were averaged within each group (Fig. 5C). One-way ANOVA indicated a main effect of group (F_(3, 45)_ = 6.928, P = 0.0006). Newman-Keuls posttest confirmed that the slope was significantly steeper in BT+ group (1.302 ± 0.272 pA/μA, n = 17) than in BT− (0.334 ± 0.132 pA/μA, n = 17, P < 0.001), shock (0.189 ± 0.106 pA/μA, n = 10, P < 0.01) and context control (0.321 ± 0.082 pA/μA, n = 5, P < 0.05) groups. Our data indicate that IA training substantially increased synaptic efficacy in the HiB5 cell-derived neurons, i.e., the newly integrated HiB5 cell derived-neurons participate in IA learning-induced synaptic potentiation.

**Figure 6:**
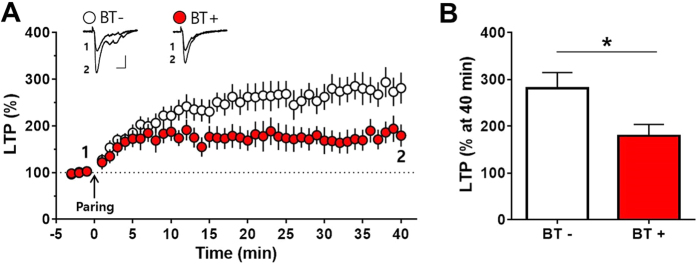
IA learning occludes pairing-induced LTP in the implanted HiB5 cell-derived neurons *ex vivo*. (**A**) Slices were prepared from homecage control (BT−) and IA-learned rats (BT+). LTP was induced by paring protocol indicated by the arrow: eighty presynaptic stimuli were delivered at 2 Hz while holding the membrane potential at +10 mV. Insets demonstrate averaged current traces of five consecutive EPSC responses at the indicated time. Scale bars, 20 ms and 50 pA. (**B**) The magnitudes of LTP were compared at 40 min after the pairing. LTP after IA learning (BT+) was significantly attenuated. *P < 0.05, unpaired Student’s t-test. Values are mean ± SEM.

To further confirm the role of the implanted HiB5 cell-derived neurons in forming the memory traces by IA learning, we tried LTP occlusion experiment. If the afferent synapses on the implanted HiB5 cell-derived neurons are already potentiated by learning, *ex vivo* LTP induction would be occluded. We prepared brain slices from BT− and BT+ groups 1 day after the behavioral treatments and compared the efficacy of paring protocol-induced LTP. As expected, the HiB5 cell-derived neurons from BT+ group showed less potentiation by the pairing protocol compared with BT− group, i.e., LTP was appreciably attenuated after learning (Fig. 6A). When the magnitudes of LTP were measured at 40 min after the pairing, they were significantly larger in slices from BT− group than BT+ group (BT−: 281.30 ± 33.51%, n = 9; BT+: 179.50 ± 24.05%, n = 8; p < 0.05, unpaired Student’s t-test; Fig. 6B). Again, the result indicates that the IA learning induces synaptic potentiation in the HiB5 cell-derived neurons, which may share similar mechanisms exploited by pairing-induced *ex vivo* LTP. It is noteworthy that IA training did not change resting membrane potential, input resistance, action potential firing frequency and more importantly, PPF (see Supplementary Fig. S3 on line). Because a change of PPF reflects the alteration of neurotransmitter release from presynaptic sites, IA training might not presynaptically strengthen synapses onto HiB5 cell-derived neurons.

**Figure 7:**
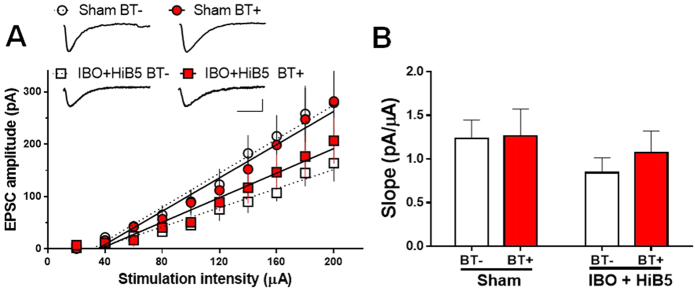
IA learning does not produce LTP at synapses onto endogenous CA1 pyramidal neurons. (**A**) Input-output relationships of EPSCs for endogenous CA1 pyramidal cells in the slices from the IBO-lesioned rats that received HiB5 cells (IBO + HiB5) and the saline-injected rats (Sham). Recordings were made in the endogenous CA1 pyramidal cells 1 day after IA learning (BT+). Homecage-control was used as a control (BT−). Insets demonstrate averaged current traces of five consecutive EPSC responses with input stimulations of 200 μA. Scale bars, 20 ms and 100 pA. (**B**) Comparison of the slopes calculated from the input-output curves shown in A. In each group, the slope of BT+ was not different from that of BT−. P > 0.05, unpaired Student’s t-test. Values are mean ± SEM.

Meanwhile, we could not observe any significant IA learning-induced enhancement of synaptic efficacy in endogenous CA1 pyramidal cells. Hippocampal slices were prepared 1 day after IA learning from either the IBO-lesioned rats that received HiB5 cells (IBO + HiB5) or the saline-injected rats (Sham). The input-output relationships for the EPSC amplitude as a function of stimulus intensity were compared in endogenous CA1 pyramidal cells from homecage control (BT−) and IA learning (BT+) (Fig. 7A). The slopes of the linear fits to the data points in each endogenous pyramidal cell were obtained and they were averaged within each group (Fig. 7B). In IBO + HiB5 group, the slope from endogenous CA1 pyramidal cells around the HiB5 cell-derived neurons was not significantly different regardless of the behavioral treatment (BT−: 0.84 ± 0.17 pA/μA, n = 7; BT+: 1.07 ± 0.24 pA/μA, n = 8; P < 0.05, unpaired Student’s t-test). In addition, IA learning made little difference in the slope from endogenous pyramidal cells from Sham group (BT−: 1.23 ± 0.21 pA/μA, n = 16; BT+: 1.26 ± 0.31 pA/μA, n = 14; P < 0.05, unpaired Student’s t-test). Therefore, IA learning-induced enhancement of synaptic efficacy may occur preferentially in the implanted HiB5 cell-derived neurons rather than endogenous CA1 pyramidal cells.

## Discussion

The hippocampus is a vulnerable structure that plays a pivotal role in cognitive process^28^,^29^. Hippocampal neuronal loss, associated with many disease states such as Alzheimer’s disease, head injury, ischemia, epilepsy and severe stress, induces deficits in learning and memory. In our lesion model, IBO injection generated about 30% loss of CA1 pyramidal cells (see Supplementary Fig. S4 on line). HiB5 cells implanted into the hippocampal alveus were found in CA1 stratum pyramidale, indicating migration of HiB5 cells toward damaged CA1 pyramidal layer (Fig. 1B,C). This suggests that these cells might be attracted by chemoattractants released from the damaged tissue. We suppose that neuronal death in CA1 area due to IBO-lesion would activate astrocytes and microglia, which results in release of cytokines. Indeed, TNFα and INFγ are known to act as chemoattractants that guide the migration of NPCs to the injured area^30^,^31^. SDF-1 is also up-regulated in astrocytes and endothelial cells in injured tissue and attracts NPCs^32^. Therefore, it is possible that HiB5 cells were guided to CA1 stratum pyramidale by chemoattractants released from the injured area.

Previously, it has been shown that immortalized NPCs implanted into the cortex or hippocampus can give rise to region-specific neurons: fully differentiated NPC-derived neurons display similar morphological features to neighboring endogenous pyramidal cells^33^,^34^,^35^,^36^,^37^. In agreement with the previous studies, implanted HiB5 cells found in CA1 stratum pyramidale appeared to differentiate into vGluT1-positive glutamatergic neurons with morphological properties of endogenous CA1 pyramidal cells (Figs 1 and 3). Besides, electrophysiological recordings demonstrated that the implanted HiB5 cells also displayed the intrinsic membrane properties identical to those in endogenous CA1 pyramidal cells (Fig. 2). Given that the implantation significantly attenuated neuronal loss in CA1 pyramidal layer (see Supplementary Fig. S4 on line), the implanted HiB5 cells-derived neurons might replace dead neurons, albeit partially. In fact, we found that a significant portion of HiB cells (~13%) survived 28 days after implantation (see Supplementary Fig. S1 on line).

Hippocampal implantation studies have reported a functional integration of implanted NPCs in the sense that excitable membranes and postsynaptic currents are detected^35^,^38^,^39^. Moreover, activity dependent short-term and long-term plasticity of afferent synapses on the implanted NPC-derived neurons have been observed^38^. We also found that the implanted HiB5 cell-derived neurons functionally integrate into the existing hippocampal circuits (Fig. 3). Morphologically, HiB5 cell-derived neurons harbored dendritic spines closely apposed to presynaptic terminals. Electrophysiologically, paired-pulse stimulations on their afferents generated evoked EPSCs that displayed PPF. More importantly, the pairing protocol resulted in LTP formation in HiB5 cell-derived neurons (Fig. 6). Thus, the implanted HiB5 cell-derived neurons appeared to have the ability to establish functional synaptic connections whose efficacy can be modified in activity-dependent manners. Consequently, we anticipated that these cells might restore damaged hippocampal circuitry, thereby recover brain function. As expected, implantation of HiB5 cells significantly improved the learning deficit in our IBO-lesion rat model, when assessed by IA learning paradigm (Fig. 4).

In the past several decades, there has been considerable evidence indicating that mechanisms mediating learning and memory involve activity-dependent changes in synaptic efficacy^14^,^15^. Indeed, experience-dependent synaptic modifications have been reported in the hippocampus^40^,^41^,^42^. More directly, it has been demonstrated that IA learning induces LTP in the hippocampus^26^. Therefore, we tested the possibility that learning induces synaptic modification in implanted HiB5-cell derived neurons. If the synaptic efficacy changes following IA learning, one can consider that implanted cell-derived neurons function properly in the context of host neural circuit by replacing lost neurons. More importantly, it will support the notion that implanted NPC-derived neurons could directly contribute to the formation of a new memory trace. Remarkably, IA learning produced synaptic strengthening at the synapses onto the HiB5 cell-derived neurons (Fig. 5), which attenuated paring protocol-induced *ex vivo* LTP (Fig. 6). However, the learning did not significantly change the strength of synapses onto the endogenous CA1 pyramidal cells (Fig. 7). At present, we do not know why the implanted HiB5 cell-derived neurons are preferentially recruited to the learning process. Nevertheless, our study supports the previous reports that newborn neurons in the adult dentate gyrus tend to be preferentially activated during spatial exploration^43^,^44^. It has also been reported that immature neurons express a more robust LTP than mature neurons in the adult dentate gyrus^45^,^46^. Our findings thus imply that information processing in the lesioned hippocampus is carried out primarily by newly incorporated young neurons derived from implanted NPCs.

Although our present study presents direct evidence of the functional synapses onto the implanted HiB5 cell-derived neurons, a functional synaptic formation in their projection sites remains to be examined. There are only a few studies demonstrating functional synaptic connectivity of implant cell-derived neurons in their axonal projection sites. Using an optogenetic approach, implanted human pluripotent stem cells-derived neurons generate inhibitory postsynaptic currents in host hippocampal neurons^47^. It has been demonstrated that light activation of ChR2 expressing spinal motor neurons derived from murine embryonic stem cells induced muscle twitches^48^. Therefore, in order to confirm the functional synaptic formation of implanted cells with host cells in their projection sites, optogenetic manipulations of implanted cells will be a powerful approach.

Many studies indicate that implanted NPCs integrate into pre-existing neural circuits, replacing lost cells, thereby functionally restore damaged brain tissues^7^,^8^,^9^. Our study is in line with this notion of cell replacement therapy. Besides this mechanism, it has been suggested that beneficial therapeutic effects of NPCs implantation on brain function are attributed to the fact that NPCs exert beneficial ‘bystander effects’ on microenvironment^49^. Indeed, implanted neural precursor cells can provide neuroprotection by secreting trophic factors and also by immune modulation^50^,^51^. It is not clear whether the behavioral improvement and the decrease of neuronal loss by the HiB5 cell implantation are mainly due to cell replacement effects or bystander effects. Regardless of the mechanism, to our best knowledge, no study has ever showed learning-induced synaptic modification in implanted NPC-derived neurons. Here we have first demonstrated that learning actually produces potentiation at the synapses onto implanted NPC-derived neurons. Therefore, we conclude that implanted NPC-derived neurons in the damaged hippocampus actively participate in learning process through LTP formation, thereby restoring defective learning and memory abilities.

## Materials and Methods

### HiB5 cell culture

A rat hippocampal progenitor cell line, HiB5 (kindly provided by Dr. R.D. McKay) which has been immortalized with a temperature sensitive SV40 T antigen^21^, was maintained in Dulbecco’s modified Eagle’s medium (DMEM) containing 10% fetal bovine serum (FBS) to passages 12–16 at 33 °C in 5% CO_2_ incubator. For the implantation, HiB5 cells were infected with GFP (or LacZ)-expressing adenovirus and labeled with fluorescence dye, DiI-C18-(3) (DiI, 60 μg/ml). HiB5 cells (2 × 10^5^ cells) were plated in a 100 mm dish 48 h before viral infection. Cells were infected with adenovirus at 5 to 50 plaque forming units (pfu) per cell in DMEM media containing 2% FBS for 2 hr at 33 °C. Cells were then labeled with fluorescence dye, DiI-C18-(3) (DiI, 60 μg/ml), for 2 hr at room temperature in darkness, followed by incubation in DMEM containing 10% FBS overnight. Before implantation, the cells were treated with PDGFB (20 μg/ml) in N2 media for 2 hr at 39 °C. After that, the cells were trypsinized, and the number of viable cells was counted in N2 media containing trypsin inhibitor by trypan blue staining. The cells were diluted at a density of 6 × 10^4^ cells/μl in the saline containing 0.7% penicillin/streptomycin and 0.5% glucose (20 mM HEPES, pH 7.2) and used for the implantation.

### Animals and surgery

Male Sprague–Dawley rats (220–250 g) were purchased from the Orient Co., a branch of Charles River Laboratories (Seoul, Korea). Rats were housed 2 or 3 per cage, allowed access to water and food ad libitum, and maintained under a constant temperature (23 ± 1 °C), humidity (60 ± 10%) and a 12 h light/dark cycle (light on 07.00–19.00 h). Animal maintenance and treatment were carried out in accordance with the Animal Care and Use Guidelines issued by Kyung Hee University, Korea. All experiments with rats were performed according to the protocols approved by the Institutional Animal Care and Use Committee of Kyung Hee University (Approved protocol No. KHUASP(SE)-13-031).

Stereotaxic injection of ibotenic acid (IBO) was performed as previously described^17^,^18^. Rats were anesthetized with equithensin (350 mM sodium pentobarbital, 250 mM chloral hydrate, 85 mM MgSO_4_, 40% prophylene glycol in 10% ethanol, 2 ml/kg). IBO (3 μl per animal, 1 mg/ml in 0.9% saline) or equal amount of saline (for Sham group) was injected into the entorhinal cortex as follows: rats were placed in a stereotaxic device (Stoelting) with the incisor bar 3.4 mm below the interaural line and the needle was positioned at an angle of 10° right to the midsaggital plane. IBO or saline was injected at 6 different locations within the entorhinal cortex (first, AP: −8.4 mm, ML: ±4.8 mm, DV: −4.3 to −4.5 mm; second, AP: −8.4 mm, ML: ±4.8 mm, DV: −2.3 to −2.5 ; third, AP: −8.8, ML: ±3.6, DV: −3.2 to 3.4). For IBO + HiB5 group, IBO-injected rats received bilateral infusions of HiB5 cells in the alveus of the hippocampus (AP: −4.3, ML: ±1.4 and DV: −2.2). Two microliters of HiB5 cells (6.0 × 10^4^ cells/μl) were injected into each site. For the non-implanted groups (IBO group and Sham group), rats received an equal amount of vehicle without cells (saline containing 0.7% penicillin/streptomycin, 0.5% glucose, 20 mM HEPES, pH 7.2) in the alveus. The syringe was left at the injected position for 10 min and then withdrawn over 5 min to minimize loss of cells from the injection site.

### Electrophysiology

Rats were decapitated, and brains were rapidly removed and placed into cold oxygenated (95% O_2_, 5% CO_2_) sucrose solution with the following composition (in mM): 175 sucrose, 20 NaCl, 3.5 KCl, 1.2 NaH_2_PO_4_, 26 NaHCO_3_, 1.3 MgCl_2_, and 11 glucose. Coronal slices of 300 μm thickness were cut with vibroslicer (Campden Instrument) and left to adapt to room temperature (21 ~ 23 °C) for 1 hr in oxygenated artificial cerebrospinal fluid (ACSF, in mM): 120 NaCl, 3.5 KCl, 1.25 NaH_2_PO_4_, 26 NaHCO_3_, 1.3 MgCl_2_, 11 glucose, and 2 CaCl_2_. The slices were then transferred to the recording chamber, where they were fully submerged, continuously perfused with ACSF at a flow rate of 1.2 ~ 1.5 ml/min, and maintained at 32 ± 1 °C.

All recordings were performed with the patch-clamp technique in whole-cell configuration, using an EPC10 amplifier (HEKA Elektronik). Patch-clamp pipettes were pulled using PP-83 (Narishige Scientific Instrument Lab.) from borosilicate glass of outer diameter 1.2 mm with a tip resistance of 3–4 MΩ when filled with internal solution. Two different internal solutions were used. For measuring passive membrane properties and firing properties, the following solution was used (in mM): 120 K-gluconate, 10 HEPES, 1 MgCl_2_, 5 NaCl, 0.2 EGTA, 2 Mg-ATP, and 0.3 Na-GTP; pH was adjusted to 7.2 with KOH. Excitatory postsynaptic currents (EPSCs) were recorded using a cesium-based solution (in mM): 120 CsCl, 10 HEPES, 1 MgCl_2_, 5 NaCl, 0.2 EGTA, 2 Mg-ATP, and 0.3 Na-GTP; pH was adjusted to 7.2 with CsOH. Biocytin (0.5%) was included in the internal solution for *post-hoc* morphological analysis. Because implanted HiB5 cells were labeled with both GFP and DiI, the implanted cells located in the hippocampal CA1 pyramidal layer were visualized using immunofluorescence and infrared differential interference contrast (IR-DIC) video microscopy with a 40× magnification water-immersion objective (BX51WI, Olympus). During slice recordings, photobleaching of fluorescent dyes sometimes hindered identifying implanted cells, especially when GFP expression was relatively low. Therefore, we switched excitation wavelengths (488 nm for GFP excitation and 549 nm for DiI, respectively) to minimize the bleaching, which helped us confidently differentiate implanted cells from endogenous cells. After whole-cell configuration, the series resistance was regularly monitored and a maximum series resistance of 15 MΩ was tolerated. Picrotoxin (100 *μ*M) was used to block GABAergic inhibitory currents for EPSC recordings. The holding potential was -70 mV for evoked EPSCs. To induce LTP, 80 presynaptic stimuli were delivered at 2 Hz while holding the membrane potential at +10 mV. The stimulus intensity was adjusted to produce EPSCs with an average amplitude of ~50 pA. This paring protocol was delivered no later than 10 min after the establishment of whole-cell recording. Data were sampled at 5 kHz and filtered at 3 kHz with Bessel filter of the amplifier. EPSCs were measured using Patcher’s Power Tools in IgorPro (Wavemetrics Inc.).

### Inhibitory avoidance learning

The inhibitory avoidance test was carried out as previously described^18^,^52^. The apparatus is a two-chambered acryl box consisting of a lighted side and a dark side separated by a trap door. Rats were habituated in the apparatus 25 and 26 days after the surgery; the rats avoid the light by entering the dark chamber through the acryl door when the light is turned on. This was repeated for two days until the rats entered the dark chamber within 20 s (Habituation). The next day, the rats were placed in the lighted chamber and when they entered the dark chamber, the trap door was closed and an electrical foot shock (1 mA) was delivered for 3 s through the grid floor (Learning). Twenty-four hours after the learning trial, the rats were placed in the lighted chamber and the latency time to enter the dark chamber was measured for 720 s (Test). To avoid possible bias, we blindly performed the experiments.

### Cell staining

Immunostaining analysis of brain slices was carried out as previously described with modifications^18^,^53^. For the identification of implanted HiB5 cells and further analysis of detailed morphological aspects, biocytin, which was included in the pipet solution during electrophysiological recording, was retrospectively processed for immunohistochemistry. The hippocampal slices (300 μm) were fixed with 4% paraformaldehyde in PBS for 4 hr at 4 °C, and then the slices were cryoprotected in 30% sucrose–PBS for over-night. Dehydrated hippocampal slices were cryosectioned at a thickness of 40 μm. Sections were stored at 4 °C in storing solution (30% glycerol, 30% ethylene glycol in PBS). The cryosectioned hippocampal slices were permeabilized in 0.5% Triton X-100 for 20 min and blocked in 15% normal serum with 3% BSA and 0.1% Triton X-100 for 2 h in free floating condition. The sections were incubated for 16 hr at 4 °C with antibodies against NeuN (dilution ratio 1:500, Chemicon), GFAP (1:750, BM), O4 (1:700, BM), GFP (1:1000, Chemicon-Millipore; 1:1500, Abcam), VGluT1 (1:400, Chemicon-Millipore), synapsin I (Syn I, 1:500, Chemicon-Millipore), glutamate (1:100, Chemicon-Millipore) and GABA (1:50, Chemicon-Millipore). Secondary antibodies conjugated with Alexa Fluor® 488 (1:700, Molecular probe) and Alexa Fluor® 546 (1:700, Molecular probe) were used. Biocytin labeled dendritic spines were visualized by Alexa Fluor® 488- or Alexa Fluor® 546-conjugated streptavidin (1:1000, Jackson immunoresearch). Immunostained cells were scanned using a confocal laser microscope (LSM510, Carl Zeiss).

For X-gal staining, animals were perfused with 0.5% glutaraldehyde in 100 mM PIPES (pH 6.9), 2 mM MgCl_2_ and 5 mM EGTA. Brains were cryosectioned at a thickness of 40 μm. Sections were then incubated in X-gal staining solution composed of 5 mM K_4_Fe(CN)_6_, 5 mM K_3_Fe(CN)_6_, 2 mM MgCl_2_, and 1 mg/ml 5-bromo-4-chloro-3-indolyl-D-Galactoside (X-gal) overnight at 37 °C. Blue X-gal signal localized only in the nucleus was counted. The numbers of LacZ-expressing HiB5 cells in all the serial sections of dorsal hippocampal area of the brain (AP: Bregam −2.30 ~ −4.52) were counted and the percentage of survived HiB5 cells out of total implanted HiB5 cells were calculated at different time points.

For Nissl (cresyl violet) staining, every fifth sections (20 μm in thickness) of the hippocampal region of the brain tissues (AP: Bregma −4.16 to −4.52 mm) were taken. Nissl-stained cells were counted in CA1 pyramidal layer and the mean number of Nissl-stained cells was normalized to that of Sham group.

### Statistics

All data are presented as mean ± SEM. Behavioral data were analyzed by one-way analysis of variance (ANOVA) followed by Newman-Keuls posttest. For electrophysiological data, statistical significance was assessed using unpaired Student’s t-test, one-way ANOVA, or two-way ANOVA, when appropriate, followed by Newman-Keuls posttest. Mean differences between groups were considered significant when P < 0.05.

## Additional Information

**How to cite this article**: Park, K. *et al.* Learning-induced synaptic potentiation in implanted neural precursor cell-derived neurons. *Sci. Rep.* **5**, 17796; doi: 10.1038/srep17796 (2015).

## Supplementary Material

Supplementary Information
