# Supplementary material for: Learning-induced synaptic potentiation in implanted neural precursor cell-derived neurons
Source: Sci Rep. 2015 Dec 4;5:17796. doi: 10.1038/srep17796 (PMC4669478; doi:10.1038/srep17796)
Supplement: Supplementary Information [file srep17796-s1.pdf]

Supplementary Information

Learning-induced synaptic potentiation in implanted neural precursor cell-derived neurons

Kyungjoon Park,[1,a] Hwon Heo,[1,a] Ma Eum Han,[1] Kyuhyun Choi,[1] Jee Hyun Yi,[1] Shin Jung Kang,[2] Yunhee Kim Kwon,[1,*] Ki Soon Shin[1,*]

[1]Department of Biology, Department of Life and Nanopharmaceutical Sciences, Kyung Hee University, Seoul, Republic of Korea; [2]Department of Molecular Biology, Sejong University, Seoul, Republic of Korea

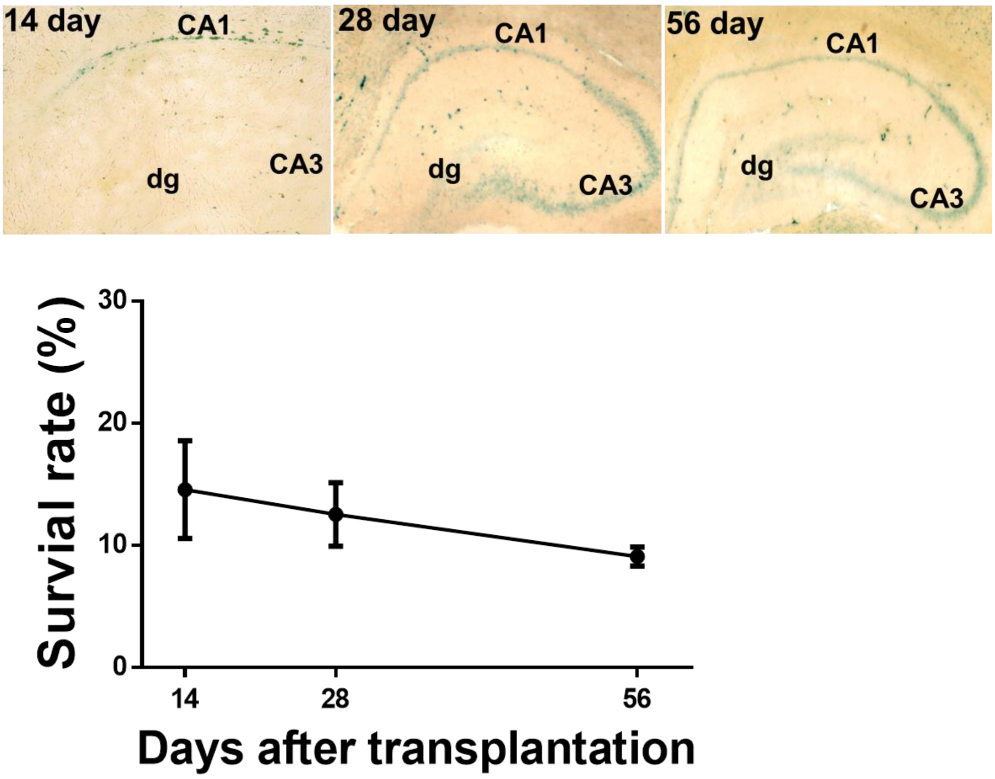

Supplementary Figure S1. Survival rate of HiB5 cells in the hippocampus 14 to 56 days after implantation. HiB5 cells were infected with LacZ-expressing adenovirus prior to implantation and visualized by X-gal staining (upper panel). The numbers of LacZ-expressing HiB5 cells in all the serial sections of hippocampal area of the brain were counted and the percentage of survived HiB5 cells out of total transplanted HiB5 cells were calculated at different time points (bottom panel; 14 days: 14.56 ± 2.00, n = 4; 28 days: 12.54 ± 0.87, n = 7; 56 days: 9.09 ± 0.78, n = 3). Bars represent mean ± SEM.

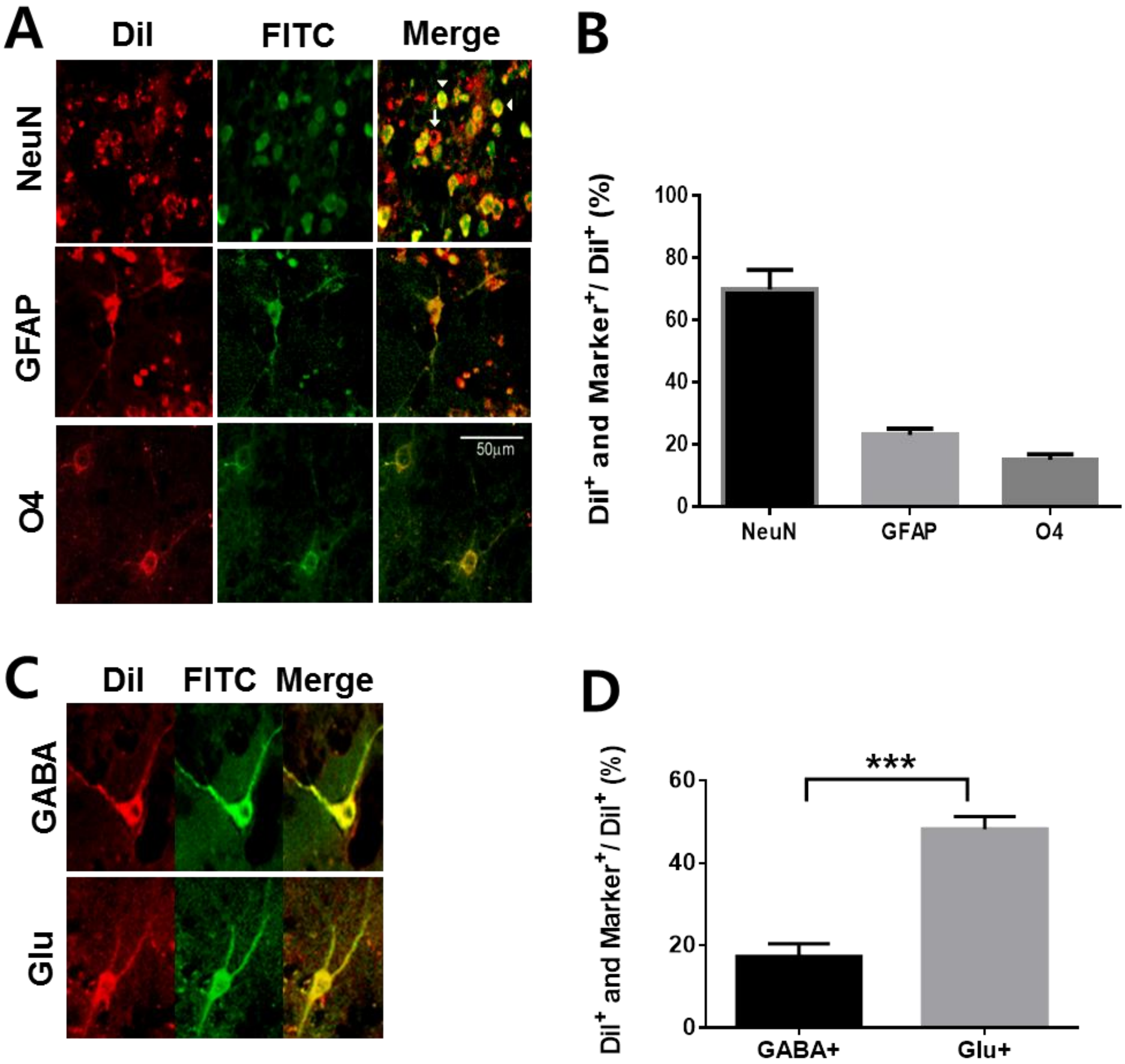

Supplementary Figure S2. Multiple differentiation potential of implanted HiB5 cells. When implanted into the alveus of the hippocampus, HiB5 cells migrated toward the entire hippocampus, including pyramidal layer and hippocampal fissure by 28 days and differentiated into three major cell types in the hippocampus. (**A**) Immunostaining of the implanted HiB5 cells (DiI-C18-(3), red) with cell type specific markers (FITC, green). Implanted HiB5 cells were able to differentiate into neurons (NeuN$^+$), astrocytes (GFAP$^+$)

or oligodendrocytes (O4$^+$). For instance, arrow heads and an arrow indicate DiI- and NeuN-double positive neurons and a DiI-positive non-neuronal cell, respectively. (**B**) Percentages of the cells double-labeled with both DiI and each cell type specific marker to total DiI-positive cells. More than sixty microscopic fields from three rats were examined for each experimental point. Each bar represents mean ± SEM (NeuN$^+$: 69.88 ± 3.65, n = 3; GFAP$^+$: 23.03 ± 1.19, n = 3; O4$^+$: 15.12 ± 1.00, n = 3). (**C**) Immunostaining of the implanted HiB5 cells (DiI, red) with specific neuronal markers (FITC, green). Implanted HiB5 cells were able to differentiate into glutamatergic neurons (Glu$^+$) and GABAergic neurons (GABA$^+$). (**D**) Percentages of the cells double-labeled with both DiI and each neuronal marker to total DiI-positive cells. More than sixty microscopic fields from three rats were examined for each experimental point. Each bar represents mean ± SEM (GABA$^+$: 17.33 ± 1.74, n = 3; Glu$^+$: 48.21 ± 1.76, n = 3).

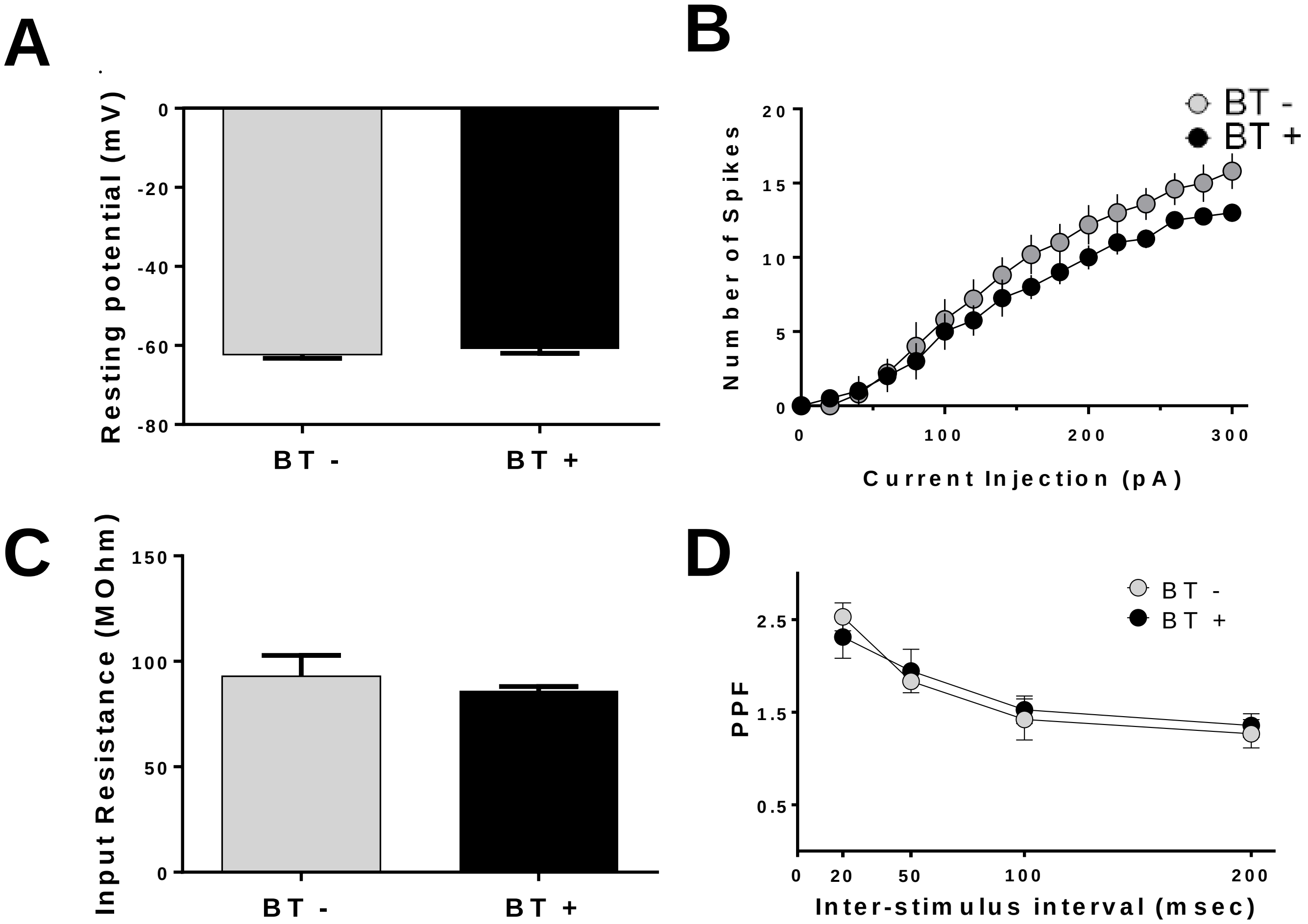

Supplementary Figure S3. IA learning did not change resting membrane potential, firing frequency of action potential, input resistance and PPF in implanted HiB5 cell-derived neurons. Pacth clamp recordings were made on HiB5 cell-derived neurons from naïve (BT-) or IA-trained rats (BT+). Values are mean ± SEM (**A**) IA learning did not change resting membrane potential (BT-: -62.28 ± 0.96 mV, n=5; BT+: -60.78 ± 1.21 mV, n=5; $P < 0.05$, unpaired Student's t-test) (**B**) IA learning did not change action potential frequency ($F_{(1,7)} = 1.072$, $P = 0.334$, repeated measure two-way ANOVA) (**C**) IA learning

did not change input resistance (BT-: 92.98 ± 9.774 Ω, n=5; BT+: 86.00 ± 2.104 Ω, n=5; P < 0.05, unpaired Student's t-test) (**D**) IA learning did not change PPF ($F_{(1,9)}$ = 0.015, P = 0.9039, repeated measures two-way ANOVA).

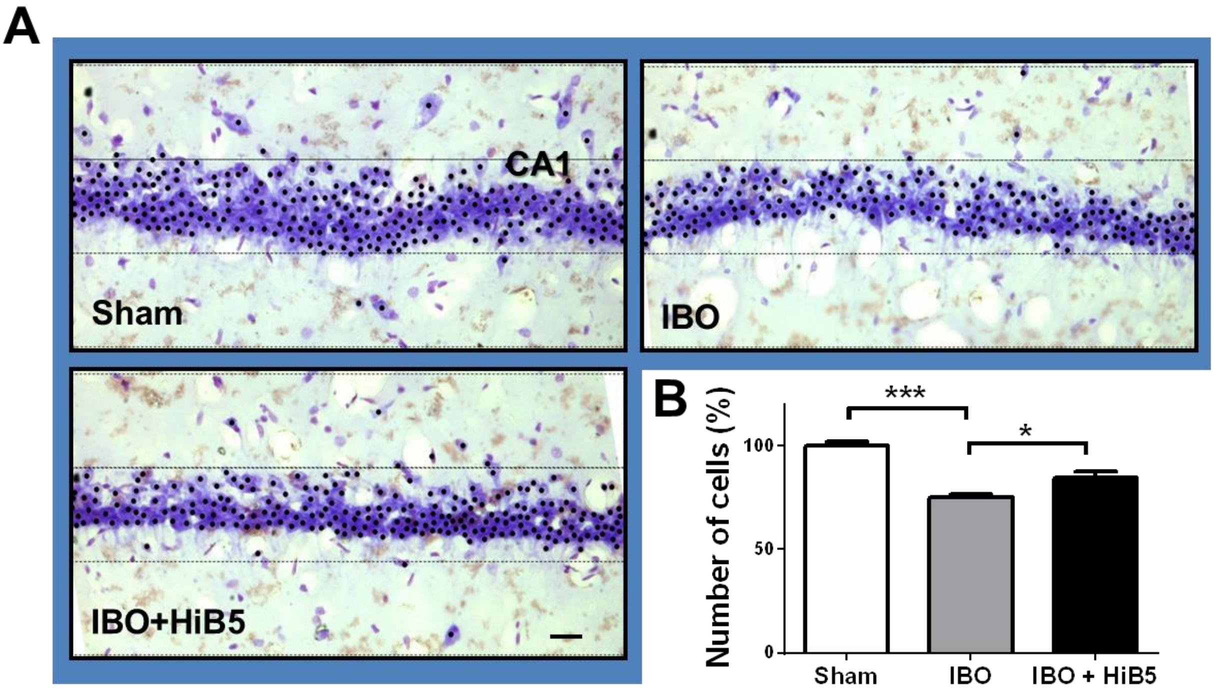

Supplementary Figure S4. Attenuation of neuronal loss by HiB5 cell implantation. (**A**) Nissl-stained cells in CA1 stratum pyramidale were shown in slices from Sham, IBO-lesioned and IBO+HiB5 rats, 28 days after surgery. Scale bar represents 50 μm. (**B**) Twenty eight days after implantation, Nissl-stained cells were counted in CA1 stratum pyramidale and the mean number of Nissle-stained cells normalized to that of Sham group was calculated. Each bar represents the mean ± SEM. A one-way ANOVA showed a significant main effect of treatments on percent number of cells in CA1 pyramidal layer ($F_{(2, 6)}$ = 34.18, P = 0.0005): while percent number of cells was decreased in IBO-lesioned rats compared with sham-operated rats (IBO: 74.84 ± 1.89, n = 3; Sham: 100 ± 1.811 s, n = 3; ***P < 0.001, *post hoc* Newman-Keuls test), HiB5 cell implantation significantly

recovered the cell number (IBO + HiB5: 84.62 ± 2.69, $n = 3$, $^*P < 0.05$, *post hoc* Newman-Keuls test).
